# Influence of *Aloe vera* (*Aloe barbadensis* M.) as an alternative to antibiotics on the growth performance, carcass characteristics and haemato‐biochemical indices of broiler chickens

**DOI:** 10.1002/vms3.1099

**Published:** 2023-03-06

**Authors:** B. Quaye, O. Opoku, V. Benante, B. Adjei‐Mensah, M. A. Amankrah, B. Ampadu, E. Awenkanab, C. C. Atuahene

**Affiliations:** ^1^ Department of Animal Science Kwame Nkrumah University of Science and Technology Kumasi Ghana; ^2^ Centre d'Excellence Régional sur les Sciences Aviaires Université of Lomé Lomé Togo

**Keywords:** *Aloe vera gel*, synthetic additive, blood, infusion, extract

## Abstract

**Background:**

Medicinal herbs as classes of additives to poultry feeds have proven to be beneficial due to their antioxidant, antimicrobial and antifungal properties.

**Objective:**

A 6‐week study was conducted to assess the effects of *Aloe vera* (*Aloe barbadensis* M.) as an alternative to antibiotics on the growth performance, carcass traits and haemato‐biochemical parameters of broiler chickens.

**Methods:**

A total of 240 unsexed commercial broiler chickens, 2 weeks old, were randomly allocated to four treatments: T1 (negative control), T2 (positive control, 1 g/L oxytetracycline), T3 (0.5% *Aloe vera* gel extract) and T4 (1% *Aloe vera* gel extract) in a completely randomised design (CRD), with six replicates of 10 birds per replicate. The *Aloe vera* gel extract was administered in fresh drinking water.

**Results:**

The results revealed across all the treatment groups, no significant (*p* > 0.05) differences were found in terms of growth performance and carcass traits. However, the mortality rate was significantly lower (*p* < 0.05) in the positive control and the *Aloe vera* groups compared to the negative control. Total cholesterol, total glucose, and high‐density lipoprotein values for the experimental groups (T3 and T4) were significantly (*p* < 0.05) lower than those of the control groups. The values for red blood cell count, haemoglobin content, mean corpuscular haemoglobin and mean corpuscular haemoglobin concentration for the birds treated with *Aloe vera gel* were significantly (*p* < 0.05) higher than those of the control groups.

**Conclusions:**

It is therefore concluded that the addition of *Aloe vera* gel extracts up to 1% in the drinking water could replace antibiotics in broiler chickens without any adverse effects on the health status and the performance of birds.

## Graphical abstract

The supplementation of *Aloe vera* gel extract as an alternative to antibiotics in the diets of broiler chickens improved white blood cell counts and reduced mortality rate.

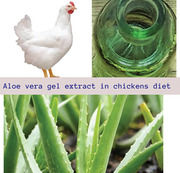

## 1 INTRODUCTION

Poultry farmers are interested in using growth promoters to raise their birds for healthy production. This has led to the use of antibiotics that can modify the intestinal microbiota and eliminate harmful bacteria which in turn improves the growth of their birds (Ayoola et al., 2020). Antibiotics as growth promoters have been very helpful in improving poultry growth, performance and feed conversion ratio (FCR), but the continued antibiotic treatment of poultry has resulted in residues in poultry products and bacterial resistance against treatment in humans (Mohamed et al., 2017). Despite rigorous withdrawal measures to prevent antibiotic residues in food, some drug residues still enter the human food chain and lead to increased antibiotic resistance (Ayoola et al., 2020). Due to such threats to human health, there have been global concerns over the use of antibiotics for growth promotion or therapeutic purposes. For these reasons, the European Union (EU) have banned the use of antibiotics as growth promoters in animal feed (Chiesa et al., 2017). Herbs, spices like ginger, pepper, garlic and various other plant extracts and essential oils are now being evaluated as an alternative to antibiotics (Abd El‐Hack et al., 2020, 2021, 2022; Diaz‐Sanchez et al., 2015).

Medicinal herbs such as *Aloe vera*, turmeric, garlic, pepper and so on as classes of additives to animals and poultry feeds have proven to be beneficial due to their antioxidant, antimicrobial and antifungal properties, (Hardy, 2002). The active compounds of herbs can be found in the form of glycosides, alkaloids, volatile oils, enzymes, organic acids and more importantly polysaccharides (Li, 2000). *Aloe vera* gel contains compounds with proven antibacterial, antiviral, antifungal, antioxidant, anti‐inflammatory, antidiabetic, immunomodulatory and wound‐healing properties (Boudreau & Beland, 2006). *Aloe vera* gel contains a primary polysaccharide known as acemannan (Boudreau & Beland 2006; Hamman, 2008), which has the ability to affect the humoral immune response and cellular immunity (Du et al., 2011). *Aloe vera* gel can withstand high acidity in the intestines of birds, ensuring its potency and impact (Darabighane & Nahashon, 2014). *Aloe vera* gel has an antibacterial activity, allowing helpful microorganisms to exhibit their effect on nutrient digestion at the expense of pathogenic bacteria activity in the gastrointestinal tract (Shokri et al., 2017). Additionally, *Aloe vera* gel is high in polyphenols and natural antioxidants, which help to prevent free radical overproduction, which causes lipid peroxidation and immune cell damage (Sharma et al., 2018). *Aloe vera* gel demonstrated antimicrobial properties against a wide range of pathogenic bacteria such as *Staphylococcus aureus* and *Escherichia coli* when Shokraneh et al. (2016) and Shilpakala et al. (2009) researched to ascertain the efficacy of *Aloe vera* as an antimicrobial agent. Despite these findings, few studies have evaluated the efficiency of *Aloe vera* on immune response in broilers (Shokraneh et al., 2016). There is a modern trend to use *Aloe vera* products as antibiotic alternatives in the poultry sector for their many benefits (Arif et al., 2022; Ebrahim et al., 2020). Hence, the study aimed to evaluate the efficacy of *Aloe vera* as an alternative to antibiotics by assessing the effects of *Aloe vera* gel extracts on growth performance, carcass traits and blood characteristics of broiler chickens.

## 2 MATERIALS AND METHODS

### 2.1 Study site and duration

The study was conducted at the Poultry Farm of the Department of Animal Science, Kwame Nkrumah University of Science and Technology Kumasi, Ghana which lies on latitude 06°41N and longitude 01°33W and with an elevation of 261.4 m above sea level. The study lasted for 6 weeks.

### 2.2 Experimental material and preparation

Fresh *Aloe vera* leaves were collected from the university farm. The leaves were cleaned thoroughly with water and the Aloe gel was extracted from the leaf by peeling off the leaves. The latex of the leaves was removed, and the gel was collected into a container. A 10% (w/v) concentrated infusion was prepared by taking 100 g of the fresh gel and putting it in 1 L of hot distilled water.

The *Aloe vera* gel was blended using an electronic blender and then poured into a glass jar. Distilled water which had a temperature of 52°C was then poured into the glass jar containing the *Aloe vera* gel mixture. The jar was shaken for 2 min to ensure thorough mixing and was then kept for 6–8 h at room temperature before use.

### 2.3 Experimental birds and design

Two hundred and forty 2‐week‐old Cobb 500 healthy birds of similar weight were selected and allocated randomly to four treatments, namely, T1, T2, T3 and T4. The four treatments were replicated six times, each with 10 birds using a completely randomised design (CRD). T1 (negative control) was provided with basal broiler diet and normal drinking water, T2 (positive control) was provided with basal diet and antibiotic (1 g/L oxytetracycline) in drinking water, and T3 and T4 were provided with basal diets and *Aloe vera* gel extracts at levels of 0.5% and 1%, respectively, in the drinking water. The standard diet contained a metabolisable energy of 3105.2 kcal/kg and a crude protein of 21.42%.

### 2.4 Housing and management

All the birds were housed in 24 deep litter pens with concrete floors. Each pen was equipped with wood shaving spread on the concrete floor to a depth of 2–5 cm with a floor space of 0.2 m^2^ per bird. Feed and water were provided ad libitum throughout the experiment. The birds were vaccinated against Gumboro and Newcastle diseases. They were also medicated against coccidiosis using a coccidiostat (amprolium).

### 2.5 Performance parameters

The chicks were weighed at the start of the experiment and then on a weekly basis after that. The chicks were weighed individually in each replicate, and weekly weight gain (g) was estimated by subtracting the initial weight from the weight at the end of the week. Weekly feed intake (g) was calculated by subtracting leftover feed from the total feed provided. By dividing feed consumed and weight gain, the feed conversion ratio (FCR) was obtained. Fresh drinking water given to the chicks was daily measured before serving. The difference between the amount of water given and the amount of water left over on a daily basis per replicate was used to calculate water intake (mL). Each pen was checked daily for the occurrence of death and the dead birds were immediately transported for post‐mortem. Mortality rate was expressed as the percentage of the number of birds that died in a pen and the total number of birds that were in the pen at the onset of the study.

$$FCR=\frac{feed\;inake}{weight\;gain},$$

$$Mortality\;rate\;\left(\%\right)=\frac{number\;of\;dead\;birds}{total\;number\;of\;birds\;per\;pen}\times 100.$$

### 2.6 Carcass analysis

At the end of this study, two birds from each replication were chosen based on the group's average body weight and humanely sacrificed by jugular vein haemorrhage following a 12‐h fast (with free access to water) for internal organ weights and carcass analyses. Birds were slaughtered, feathers plucked off and eviscerated. The carcass was cut into many pieces (breast, back, thigh, drumstick, both wings, neck and shank). The internal organs weight, dressed weight, breast, thigh, drumstick, back, wing, neck and shank were expressed as a percentage of the live weight using the formulae:

$$Primal\;cut\;\left(\%\right)=\frac{\;weight\;of\;primal\;cut}{live\;weight}\times 100,$$

$$\mathrm{Dressed}\;\left(\;\%\right)=\frac{Dressed\;weight}{\;live\;weight}\times 100,$$

$$\mathrm{Internal}\;\mathrm{organ}\;\left(\;\%\right)=\frac{Organ\;weight}{live\;weight}\times 100.$$

### 2.7 Blood analysis

Blood samples were taken from two birds from each replication at random. Blood samples were taken from the jugular vein using a 2 mL sterile needle and syringe into anticoagulant (EDTA) bottles after the feed was withdrawn. Total red blood cells (RBC), haemoglobin (HB), lymphocytes, mean corpuscular volume (MCV), thrombocytes s, haematocrit (HCT) and white blood cells (WBC) were measured using a Haematological Auto Analyzer (AR 6300, ARI Medical Technology Co., Ltd.), and biochemical factors were measured using a ‘FlexorJnr autoanalyzer’ (Vital Scientific, NV). In a macro centrifuge, blood samples obtained for serum biochemistry were centrifuged for 10 min at 3000 rpm (revolutions per minute) to create a serum for biochemical examination. The serum samples were stored frozen until they could be analysed further. Before analysis, the frozen serum samples were allowed to thaw, and the thawed serum was pipetted into dry clean tubes and refrigerated at –20°C. Total protein, total cholesterol, triglycerides, high‐density lipoprotein (HDL) and low‐density lipoprotein (LDL) were among the assay parameters assessed by serum enzyme‐linked immunosorbent assay (ELISA) (LDL).

### 2.8 Statistical analysis

The entire data collected on performance (feed intake, body weight gain, water intake and FCR), serum biochemistry (total cholesterol, total protein, triglycerides, LDL and HDL) and haematology (WBC, RBC, Hb, HCT, lymphocytes, thrombocytes and MCV) were computed and subjected to the analysis of variance (ANOVA) using Minitab Software (Version 18.1) at *p* < 0.05. Differences between treatment means were separated using the Tukey's test.

## 3 RESULTS

### 3.1 Growth performance

Table 1 shows the growth performance of the birds supplemented with *Aloe vera* extract. No significant (*p* > 0.05) differences were recorded for feed intake, water intake, body weight, body weight gain and FCR across all the treatment groups. However, the negative control group recorded a statistically (*p* < 0.05) higher mortality rate when compared to the other treatment groups. The post‐mortem examination revealed that necrotic enteritis was the cause of the death. Patches of necrotic tissue were found on the intestinal epithelium.

**TABLE 1:** Effects of *Aloe vera* gel extracts on the performance of broiler chickens during 2–8 weeks.

	Treatments					
Parameters	T1	T2	T3	T4	SEM	*p* Value
Initial weight (g)	222.5	207.5	254.2	236.7	5.97	0.526
Final weight (g)	1968	1929	2161	2102	23.8	0.427
Total feed intake (g)	3714	4013	4352	3952	101.63	0.611
Daily feed intake (g)	88.42	95.54	103.62	94.10	1.14	0.611
Total water intake (mL)	8759	9236	11061	9418	250.50	0.443
Daily water intake (mL)	208.5	219.9	263.3	224.2	8.91	0.443
Total weight gain (g)	1855	1826	1995	1853	28.16	0.735
Daily weight gain (g)	44.18	43.47	47.50	44.13	0.51	0.735
FCR	1.987	2.204	2.193	2.134	0.02	0.687
Mortality rate (%)	6^a^	2^b^	2^b^	2^b^	0.65	0.0001

^a,b^ Means within the same rows but not sharing the same superscripts are significantly (*p* < 0.05) different. FCR, feed conversion ratio.

**TABLE 2:** Carcass parameters and internal organs of birds supplemented with *Aloe vera*.

	Treatments					
Parameters	T1	T2	T3	T4	SEM	*p* Value
Live weight (g)	1968	1929	2161	2102	23.8	0.427
Defeathered weight (%)	92.63	91.19	91.84	89.40	2.23	0.577
Dressed weight (%)	78.00	77.25	78.11	76.13	2.16	0.786
Breast weight (%)	20.36	21.31	20.38	19.62	0.69	0.192
Thigh weight (%)	11.00	9.43	11.34	10.31	0.71	0.104
Drumstick weight (%)	9.83	10.45	10.19	9.17	0.74	0.395
Wings weight (%)	3.90	4.24	3.82	3.81	0.26	0.348
Neck weight (%)	4.23	4.10	4.70	4.50	0.60	0.732
Shank weight (%)	3.73	3.51	3.73	3.61	0.29	0.552
Heart weight (%)	0.54	0.48	0.54	0.53	0.08	0.833
Liver weight (%)	2.34	2.12	1.93	1.87	0.19	0.143
Full gizzard weight (%)	3.13	2.84	3.00	3.25	0.34	0.652
Empty gizzard weight (%)	2.28	2.10	2.35	2.35	0.24	0.677
Full intestine weight (%)	8.50	7.52	7.65	7.46	1.01	0.722
Empty intestine weight (%)	6.00	5.13	5.40	5.31	0.45	0.297

### 3.2 Carcass characteristics and internal organs weight

Table 2 presents the relative carcass characteristics and relative internal organs weights. All the carcass parameters and the weight of the organs did not differ (*p* > 0.05) across the treatment groups.

**TABLE 3:** Effect of *Aloe vera* infusion in the drinking water on blood biochemical parameters of broilers.

	Treatments					
Parameters	**T1**	**T2**	**T3**	**T4**	SEM	*p* Value
Total cholesterol (mmol/dL)	3.69^a^	2.90^b^	2.70^c^	2.70^c^	0.12	<0.0001
Triglyceride (mmol/dL)	0.89^a^	0.80^b^	0.69^c^	0.41^d^	0.10	<0.0001
High‐density lipoprotein (mmol/dL)	1.40^b^	1.40^b^	1.40^b^	1.69^a^	0.073	<0.0001
Low‐density lipoprotein (mmol/dL)	1.59^a^	1.71^b,c^	1.02^c^	1.10^b^	0.12	<0.0001

^a,b,c,d^ Means within the same rows but not sharing the same superscript are significantly different (*p* < 0.05).

### 3.3 Blood indices

The effect of *Aloe vera* on the blood biochemical parameters of the birds is shown in Table 3. The values of total cholesterol were significantly (*p* < 0.05) lower in the *Aloe vera*‐supplemented groups than that in the control groups. The level of triglyceride in the blood of the *Aloe vera*‐treated birds decreased (*p* < 0.05) as the level of the supplementation increased. The *Aloe vera* treatment groups had lower (*p* < 0.05) levels of triglycerides when compared to the control groups although, among the control groups, the positive control group recorded lower levels (*p* < 0.05). For HDL levels, the 1% *Aloe vera*‐supplemented group was significantly (*p* < 0.05) superior to the other treatment groups. Additionally, the levels of LDL in the *Aloe vera*‐supplemented groups were statistically reduced (*p* < 0.05) when compared to that of the negative control group.

The impact of *Aloe vera* supplementation on haematological parameters is presented in Table 4. Red blood cell count was higher (*p* < 0.05) in the *Aloe vera* treatment groups (T3 and T4) as compared to those of the control groups, even though, among the control groups, the positive control was better (*p* < 0.05). The birds giving 0.5% *Aloe vera* treatment recorded the highest (*p* < 0.05) white blood cell count followed by T2 and T4 with T1 recording the lowest (*p* < 0.05). Haemoglobin and HCT levels were significantly (*p* < 0.05) superior in the *Aloe vera* treatment groups compared to the control groups. Mean corpuscular volume increased (*p* < 0.05) as the level of *Aloe vera* increased but this was appreciably low (*p* < 0.05) relative to the negative control. The values of MCH and MCHC were statistically superior (*p* < 0.05) in the *Aloe vera* groups when compared with the control groups. The thrombocyte level in the positive control group was significantly superior to all the other treatments but thrombocyte levels decreased (*p* < 0.05) as the level of *Aloe vera* increased.

**TABLE 4:** Effect of *Aloe vera* infusion in the drinking water on blood haematological parameters of broilers.

	Treatments					
Parameters	T1	T2	T3	T4	SEM	*p* Value
Red blood cell (10^6^/μL)	2.19^c^	2.39^b^	2.59^a^	2.65^a^	010	0.0001
White blood cell (10^4^/μL)	1.74^d^	1.98^b^	2.01^a^	1.98^b^	0.063	0.0001
Haemoglobin (g/dL)	8.10^d^	8.50^c^	9.40^b^	9.90^a^	0.41	0.0001
HCT (%)	29.50^d^	31.00^c^	33.20^b^	34.40^a^	1.10	0.0001
MCV(FL)	134.70^a^	127.00^d^	128.20^c^	129.80^b^	1.69	0.0001
MCH (pg)	34.00^d^	36.80^c^	37.30^b^	37.40^a^	0.80	0.0001
MCHC (g/dL)	27.50^d^	27.90^c^	28.30^b^	28.80^a^	0.29	0.0001
Thrombocytes (10^3^/μL)	7.00^d^	14.00^a^	13.00^b^	8.00^c^	1.76	0.0001

^a,b,c,d^ Means within the same rows but not sharing the same superscript are significantly different (*p* < 0.05). HCT, haematocrit; MCH, mean corpuscular haemoglobin; MCHC, mean corpuscular haemoglobin concentration; MCV, mean corpuscular volume.

## 4 DISCUSSION

The influence of *Aloe vera* on the growth performance of the birds revealed no significant difference. The absence of significant differences in this study as far as feed intake is concerned, suggests that the *Aloe vera* gel extract did not pose any negative effect on feed intake. This finding agrees with the findings of Shokraneh et al. (2016) who also did not find differences in feed intake after giving broiler birds *Aloe vera* extract supplements. There were no significant differences among the various treatments for water intake. This result is consistent with the findings of Online et al. (2017) who also recorded no significant effect on water intake by *Aloe vera* gel extracts supplementation in broiler drinking water. However, Shokraneh et al. (2016) recorded an increase in water consumption with a high level of *Aloe vera* extract supplementation. This increase in water intake reported by the authors could be due to environmental influences or the fact that higher inclusion than the levels in this study was used. Furthermore, the weight gain of broiler birds given the control diets was not different from those given the *Aloe vera* gel extract supplementation. The result is consistent with the findings of Jamir et al. (2019) and Online et al. (2017) who also realised in their separate studies that the supplementation of *Aloe vera* irrespective of the levels did not show any significant impact on the performance of broiler chicken in terms of body weight gain. Feed efficiency was not affected by *Aloe vera* supplementation. This finding aligns with that of Darabighane et al. (2011) who observed no significant differences between the antibiotic group and the *Aloe vera* group in terms of FCR. Contrary to this finding, Jamir et al. (2019) observed a significant improvement in FCR among the birds given 1.0 g/kg *Aloe vera* in feed as compared to the control group.

The negative control recorded the highest mortality rate compared to the other treatment groups. The differences can be attributed to the antibacterial, antioxidant and antifungal properties of *Aloe vera* gel extracts, which helped the birds in the *Aloe vera* and antibiotics groups to fight against disease infection. Shokri et al. (2017) observed that *Aloe vera* is capable of boosting the bird's immune system, and this could help the birds in fighting against infections. Likewise, Rajasekaran et al. (2005) found in their study that *Aloe vera* is capable of improving birds’ immunity and promoting cell repair. Darabighane and Nahashon (2014) recorded an increase in white blood cell count for broilers treated with *Aloe vera* gel. White blood cells help the birds in fighting against infection; hence, this could explain the low mortality rate of birds treated with the *Aloe vera* gel extracts.

The supplementation of *Aloe vera* did not influence the carcass traits and the weight of internal organs. This observation supports the results of Shokraneh et al. (2016) who found that carcass yield and internal organs weights were not significantly affected by *Aloe vera* gel extracts supplementation. However, Mohamed et al. (2017) reported significant improvement in dress weight and intestinal organs weights of broilers treated with extracts of *Aloe vera* gel. The disparities could be attributed to the proportion of the *Aloe vera* extract used and the mode of incorporation.

In the present study, total serum cholesterol, triglycerides, and LDL levels significantly decreased among the birds that received 0.5% and 1% *Aloe vera* gel in drinking water compared to those in the control treatment. The level of HDL was higher in birds that received 1% *Aloe vera* treatment as compared to the other treatment groups. According to Beppu et al. (2006), *Aloe vera* gel has acemannan, which is the main polysaccharide that can modify blood cholesterol by regulating fat metabolism in the liver. This function of the polysaccharide may be responsible for the observed decrease in cholesterol, triglycerides, and LDL among birds that received *Aloe vera* gel extract. Comparably, this present finding agrees with Misawa et al. (2012) who realised that *Aloe vera* could reduce the level of lipids in the blood by enhancing the sensitivity of cells to insulin. Taraneh (2016) also observed in their study that birds given 3% of *Aloe vera* solution in drinking water exhibit decreased uric acid, cholesterol, low‐density lipoprotein and triglyceride concentrations.

Higher levels of RBC count were recorded in the *Aloe vera*‐supplemented group compared to the control groups. This finding is in line with the finding of Shokri et al. (2017) who reported that feeding birds with *Aloe vera* improve the RBC and haemoglobin values of broilers. Iji et al. (2010) also reported that the erythropoietin effects of *Aloe vera* extract on haemopoietic cells in bone marrow can improve red blood cell production. The birds supplemented with *Aloe vera* had significantly higher haemoglobin concentration, HCT, MCH and MCHC as compared to the birds in the control groups. The increase in haemoglobin concentrations as demonstrated in this study could be attributed to essential vitamins such as thiamine, riboflavin and folic acid and essential and nonessential amino acids, which are primarily required for haemoglobin synthesis (Hamman, 2008). Polysaccharides in *Aloe vera* gel had been reported to increase erythropoiesis (Channa et al. 2014). Shokri et al. (2017) reported higher levels of haemoglobin, HCT, MCH and MCHC in birds supplemented with *Aloe vera*. Significantly superior values of MCV were recorded in the negative control group as compared to the other treatment groups. Furthermore, the positive control had a higher concentration of blood thrombocytes relative to the other treatment groups. The findings on MCV in this present study are in contrast to the report of Singh et al. (2013) who found no significant difference between the birds' given *Aloe vera* juice in drinking water and the control group.

## 5 CONCLUSION

Conclusively, the addition of *Aloe vera* gel extracts up to 1% in the drinking water could replace antibiotics in broiler chickens without any adverse effects on the health status and the performance of birds.

## AUTHOR CONTRIBUTIONS

Quaye, Bernard: formal analysis, investigation, methodology, validation, visualization, writing – original draft. Opoku, Obed: formal analysis, investigation, supervision, validation, visualization, writing – original draft. Benante, Victor: methodology, resources, software, supervision. Adjei Mensah, Benjamin: data curation, validation, writing – original draft, writing – review & editing. Amankrah, Michael Adu: data curation, investigation, methodology, validation, writing – original draft. Ampadu, Brace: conceptualization, investigation, methodology, project administration, visualization, writing – review & editing. Awenkanab, Evans: conceptualization, investigation, methodology, resources, software, visualization, writing – original draft. Atuahene, Comfort Charity: conceptualization, methodology, supervision, validation, writing – review & editing.

## CONFLICT OF INTEREST STATEMENT

The authors declare there is no conflict of interest.

## FUNDING INFORMATION

The authors declare that they have no financial support from any organisation or entirely about the content of the manuscript.

### ETHICS STATEMENT

All experimental protocols were reviewed and approved by the Ethics Committee of the KNUST, Kumasi, Ghana, a branch of the National Ethics Committee for the control and supervision of experiments on animals.

### PEER REVIEW

The peer review history for this article is available at https://publons.com/publon/10.1002/vms3.1099.

## Data Availability

The data will be provided upon reasonable request.
